# A comprehensive comparison of PARP inhibitors as maintenance therapy in platinum-sensitive recurrent ovarian cancer: a systematic review and network meta-analysis

**DOI:** 10.1186/s13048-025-01599-1

**Published:** 2025-01-30

**Authors:** Shiya Ji, Lu Chen, Yebo Yu, Xupeng Chen, Liwen Wei, Lili Gou, Cheng Shi, Susu Zhuang

**Affiliations:** 1 Department of Health Education, Nanjing Municipal Center for Disease Control and Prevention, No.3, Zizhulin Road, Nanjing, Jiangsu Province 210003 China; 2 Clinical Medicine College, Yangzhou University, Yangzhou, China; 3 Department of Social Medicine and Health Education, School of Public Health, Peking University, Beijing, China

**Keywords:** Network meta-analysis, Ovarian cancer, Poly (ADP-ribose) polymerase inhibitor, Platinum-sensitive recurrent ovarian cancer, Overall survival, Progression-free survival

## Abstract

**Background:**

PARP inhibitors (PARPis) have shown promising effectiveness for ovarian cancer. This network meta-analysis (PROSPERO registration number CRD42024503390) comprehensively evaluated the effectiveness and safety of PARPis in platinum-sensitive recurrent ovarian cancer (PSROC).

**Methods:**

Articles published before January 6, 2024 were obtained from electronic databases. The study assessed and compared survival outcomes including overall survival (OS), progression-free survival (PFS), second progression-free survival (PFS2), time to first subsequent treatment (TFST), time to second subsequent treatment (TSST), and chemotherapy-free interval (CFI). Additionally, safety outcomes were investigated, specifically focusing on grade 3–4 treatment-emergent adverse effects (TEAEs). The evaluation of OS and PFS was also conducted based on the BRCA and HRD (homologous recombination deficiency) statuses.

**Results:**

Six randomized controlled trials were examined and the four PARPis (olaparib, niraparib, rucaparib and fuluzolparib) have been found to significantly increase the PFS in entire population as well as in subgroups of HRD and BRCAm (BRCA mutation). Only olaparib demonstrated a substantial improvement in OS compared to placebo in entire population (hazard ratio [HR] 0.73; 95% confidence interval [CI] 0.60–0.90), as well as in the subgroup of BRCAm. All analyzed PARPis had significant efficacy in prolonging PFS2, TFST, TSST and CFI. For safety concerns, PARPis could significantly increase incidence of TEAEs (grade3-4), while olaparib had least haematological TEAEs (grade3-4) events compared to other PARPis.

**Conclusion:**

All included PARPis showed various degrees of benefit in survival outcomes and safety profile was acceptable for PSROC patients. Among them olaparib had the best performance in both efficacy and safety.

**Supplementary Information:**

The online version contains supplementary material available at 10.1186/s13048-025-01599-1.

## Introduction

Ovarian cancer is a highly lethal gynecologic malignancy that is associated with a poor prognosis. According to GLOBOCAN, ovarian cancer was the third most common gynecologic cancer globally in 2020, accounting for about 314,000 new cases and 207,000 deaths in the certain year [1]. Unlike other female cancers that exhibit early warning signs, the symptoms of ovarian cancer are not specific. Therefore, ovarian cancer is typically detected in advanced stages [2]. For the advanced patients, first-line treatment has evolved and normally includes debulking surgery combined with platinum-based chemotherapy, while 70–80% of them will experience a recurrence within two years [3] and the five-year survival rate is 29% [4]. The prognosis and treatment plan for women with recurrent ovarian cancer (ROC) depend on how well they respond to platinum-based chemotherapy and how long it has been since their last platinum-based therapy. Response to platinum-based chemotherapy and the progression/relapse-free interval from the last platinum-based therapy determine the prognosis and treatment strategy for ROC patients. A patient is considered as “platinum-sensitive” if he/she relapses more than 6 months after the end of the previous platinum-based therapy; if less than 6 months, he/she is categorized as “platinum-resistant” and is not eligible for subsequent platinum-based chemotherapy [5]. In contrast to the latter, platinum-sensitive patients are more prevalent and respond more sensitive to treatment with better prognoses, which is becoming a focus for novel treatments [6].

Recent evidence indicated that Poly (adenosine diphosphate [ADP]-ribose) polymerase (PARP) inhibitors (PARPis) have shown efficacy in treating ovarian cancer, especially when administered for maintenance treatment of platinum-sensitive recurrent ovarian cancer (PSROC) [7]. PARPis exploit faulty DNA repair mechanisms through synthetic lethality, leading to genomic instability and tumor cell death [8]. Homologous recombination repair (HRR) is the primary pathway for double-stranded DNA repair, and if patients have HR pathway defects (HRDs), such as mutations in the BRCA1 or BRCA 2 genes, they become more responsive to PARPi therapies through a synthetic lethal process [9]. BRCA1/2 mutations (BRCAm) are present in about 22% of ovarian cancer patients, with 15% of them having germline mutations (gBRCAm) and 7% having somatic mutations (sBRCAm) [10]. Differences in BRCA mutations and HRD status have varying impacts on the efficacy of PARPis [11]. Consequently, developing personalized treatment strategies for these patients remains a significant challenge in current clinical practice. Three PARPis, olaparib, rucaparib and niraparib, have already been approved by the U.S. Food and Drug Administration (FDA) in treating ovarian cancer and are available for patients [12], with different clinical indications and toxicity profiles, and were recommended as maintenance therapy for PSROC by authoritative guidelines like ASCO [13] and NCCN [14] in recent years.

Prior studies [15, 16] have verified the substantial impact of three PARPis on enhancing progression-free survival (PFS) in patients with ROC. Nevertheless, specific analyses of overall survival (OS) are lacking due to insufficient follow-up time, which limits the ability to assess the long-term effects of PARP inhibitors. Additionally, pooled analyses of other survival outcomes besides OS and PFS remain scarce, highlighting a critical gap in evaluating the comprehensive benefits and risks associated with these therapies. Fuzuloparib has been approved for the maintenance treatment of PSROC in China, but its efficacy and safety compared with other three PARPis have not been fully reported [17].

In this study, we performed a network meta-analysis (NMA) on these four PARPis in terms of survival outcomes and adverse effects such as treatment-emergent adverse events (TEAEs) to provide evidence for more objective and logical treatment methods. The objectives of this NMA are: (1) To comprehensively assess different PARPis in the maintenance treatment of PSROC patients; (2) To compare the differences in effectiveness and safety between different PARPis for patient individualized treatment; (3) To further explore the impact of patient subgroup characteristics (status of HRD and BRCA mutation) on the efficacy of PARP inhibitors.

## Methodology

The present study adhered rigorously to the guidelines of the Preferred Reporting Items for Systematic Reviews and Meta-Analyses (PRISMA) 2020 [18]. The research was conceptualized and executed in adherence to the guidelines by the Cochrane Handbook for Systematic Reviews of Intervention [19]. The registration number of this NMA on PROSPERO is CRD42024503390.

### Search strategy

Two researchers (SY-J and L-C) conducted separate searches of various databases, including PubMed, Web of Science, Cochrane Library, Embase, and Chinese databases such as Wanfang, CNKI, and Sinomed. The search covered publications published until January 6, 2024, without any language limitations. In addition, we thoroughly went through the reference lists of all original research and evaluated papers to find additional references. The MeSH/Emtree terms “ovarian cancer,” “randomized controlled trials,” “parp inhibitor,” and “placebo” were used for the search, and the search algorithm was adjusted to include the pertinent free terms specific to each database. Disputes over the eligibility of full-text articles were resolved by arbitration or discussion with a third reviewer (YB-Y). Supplementary Material Table S1 provides the search techniques utilized across various databases.

### Inclusion criteria

The screening process for the retrieved articles was conducted in accordance with the specified inclusion criteria: (1) randomized controlled trials (RCTs); (2) participants were at least 18 years old and diagnosed with PSROC; (3) participants had already undergone no less than two lines of platinum-based chemotherapy under their belts, and their most recent regimen had resulted in either a complete or partial response.; (4) the treatment group received maintenance therapy with PARPis, while the control group received placebo; (5) the studies reported survival outcomes as well as adverse effects.

### Exclusion criteria

Articles matching the following exclusion criteria were not included: (1) participants had undergone any treatment with PARPis prior to participating in the research; (2) irrelevant outcomes (not survival outcomes or adverse effects); (3) review articles, clinical protocols, case reports, comments, animal tests, in vitro tests, and not RCTs; (4) inadequate or unavailable data; (5) the trials that were included did not fit the diagnostic parameters.

### Data extraction and quality assessment

Two investigators (SY-J and L-C) independently gathered essential content from the articles included and using ROB2 [20] to evaluate the risk of bias of trials. All disagreements were handled by the third reviewer (YB-Y). These variables were collected: year of publication, name of the first author, number of participants, age, median follow-up period, therapeutic medicines used, therapeutic dose, hazard ratios (HRs) and 95% confidence intervals (CIs) of survival outcomes, frequency of adverse events and other information needed.

### Outcomes of interest and definitions

The main outcome measure was OS, defined as the duration from the moment of random assignment to the occurrence of death. The secondary goals assessed in this study were survival outcomes, specifically PFS, second progression-free survival (PFS2), time to first subsequent therapy (TFST), time to second subsequent therapy (TSST), and chemotherapy-free interval (CFI). PFS denotes the duration between the random assignment and the occurrence of disease progression or death, whichever happened first. PFS2 is the interval between randomization and the occurrence of the disease’s second progression. TFST is the time from randomization to the first subsequent therapy or death, TSST is the time to the second subsequent therapy or death. CFI is defined as the interval between the end of the previous platinum-based regimen and the innitiation of the subsequent chemotherapy. Safety outcomes include TEAEs (grade 3–4), TEAEs (grade 3–4) leading to treatment discontinuation, and haematological TEATs (grade 3–4). The grade 3 or 4 adverse events were evaluated by the Common Terminology Criteria for Adverse Events (CTCAE). Severe and potentially fatal toxicity is indicated by adverse outcomes of grade 3–4 [21]. Anaemia in our study refers to those population with anaemia as well as decreases in the red blood cell count, haemoglobin level or haematocrit. Leukopenia in our study refers to those with leukopenia and decreases in the white blood cell count. Thrombocytopenia in our study refers to those with thrombocytopenia and decreases in the blood platelet. Neutropenia in our study refers to those with neutropenia, febrile neutropenia, neutropenic sepsis, or decreases in the neutrophil count.

### Data analysis

We conducted the NMA under the frequentist model by using the software “R4.3.2” with the package “netmeta” [22]. To display interventions and comparisons, network plots were generated. The node in each plot denotes the intervention regimen, and the line in each plot denotes a direct comparison. We measured the frequency of TEAEs in the NMA using odd ratios (ORs) and 95% CIs. The survival outcomes of OS, PFS, PFS2, CFI, TFST, and TSST were quantified using HRs and their 95% CIs. The random-effects model was applied in this NMA for mixed treatment comparisons. The Q test, proposed to be a generalization of Cochran’s test, was applied to assess heterogeneity, or inconsistency between and within designs. Forest plots were conducted to display direct and indirect comparisons among various arms. Efficacy of all arms were ranked by the P-score. The best regime has a P-score of 100%, while the worst has a score of 0%. All tests were two-sided and had a significance threshold of *p* < 0.05.

## Results

### Literature search results

**Fig. 1:**
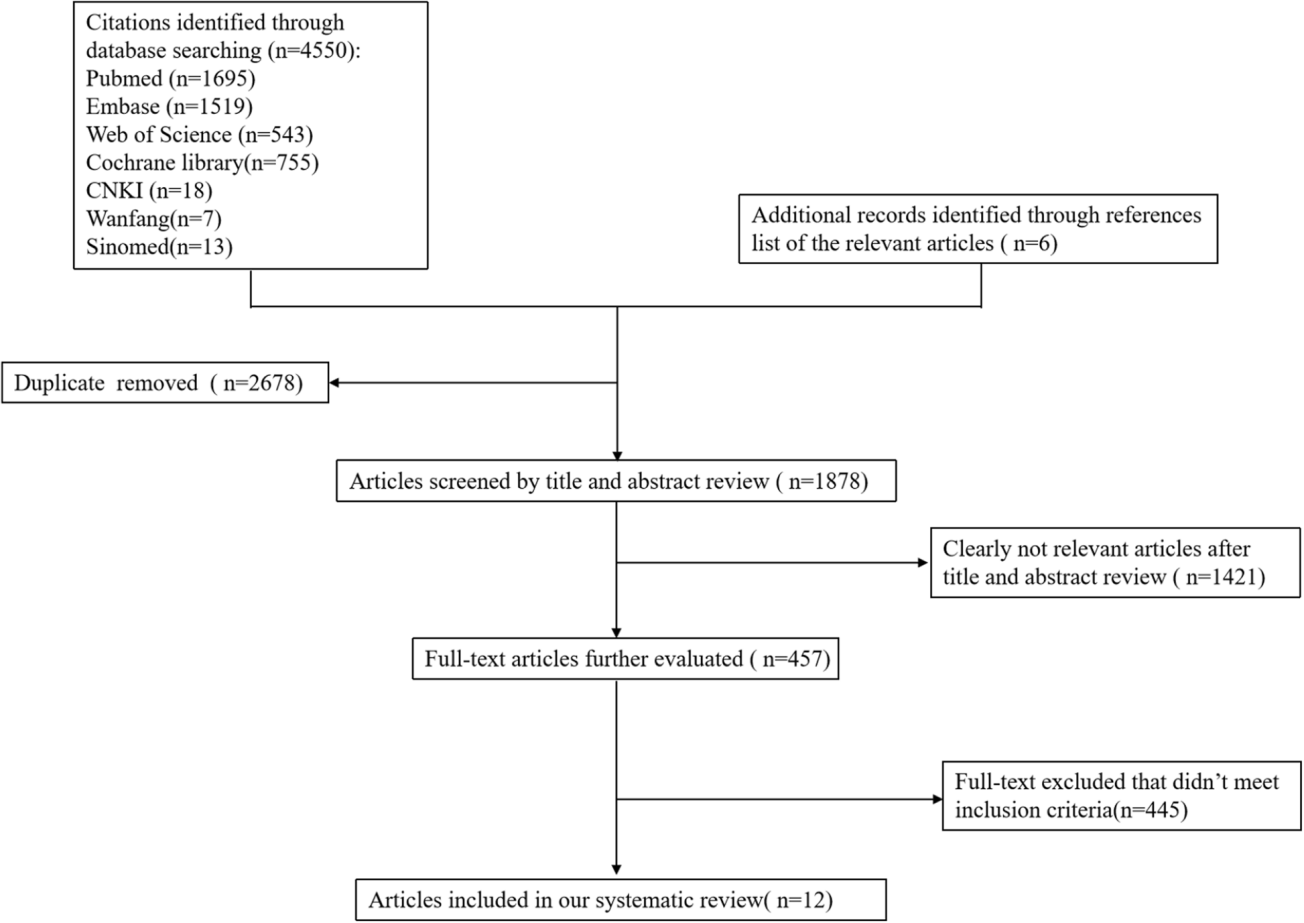
Study flow diagram

During the preliminary inspection, a total of 4556 pertinent papers were obtained utilizing the study’s search methodology. After utilizing Endnote X9 software to remove 2678 duplicate citations, we acquired the titles and abstracts for 1878 citations. Next, we evaluated the titles and abstracts to eliminate 1421. Then, 457 relevant publications with full text were evaluated for eligibility and 445 articles were precluded that did not meet inclusion criteria. 12 articles for six RCTs were included in this NMA [23–34]. The search flow diagram is displayed in Fig. 1.

### Characteristics of included studies

The current analysis includes six RCTs with 2194 patients. Table 1 summarizes the characteristics of the trials included. Despite various treatments of included studies, the baseline characteristics of all trials are comparable. It should be noted the trial NOVA (NCT01847274) had two cohorts by mutation or not of gBRCA and all endpoints were reported separately, while its two cohorts were conducted as two independent trials in the NMA. The trial SOLO2(NCT01874353) only included population with BRCA mutation. The network of trials for various outcomes is displayed in graphical network structures (Supplementary Material Fig. S1). Every circular node stands for a certain kind of regime. The quantity of studies that conduct direct comparisons is reflected in the width of the lines.

**Table 1:** Characteristics of the trials included in the network meta-analysis

Author	Year	Title of research	Location	Treatment	Sample size	Age (years)	Survival outcomes	Safety outcomes (grade 3–4)	Follow-up(months)	Registered number
Ning et al.	2022	FZOCUS-2	China, multicenter	Fuzuloparib 150 mg twice daily	167	54 (34–75)	PFS^a^ (gBRCAm^b^/entire population) CFI^c^	TEAEs^d^ TEAEs leading to treatment discontinuation TEAEs of anaemia/ leukopenia/ thrombocytopenia/ neutropenia	8.5 (open)	NCT03863860
				Placebo	85	54 (29–73)				
Wu et al.	2021	NORA	China, multicenter	Niraparib 300 mg once daily	177	53 (35–78)	OS^e^ (gBRCAm/ entire population) PFS (gBRCAm/entire population) CFI/TFST^f^	TEAEs TEAEs leading to treatment discontinuation TEAEs of anaemia	15.8 (open)	NCT03705156
				Placebo	88	55 (38–72)				
Coleman et al.	2017	ARIEL3	International, multicenter	Rucaparib 600 mg twice daily	375	61 (53–67)	OS (gBRCAm/HRDp^g^/entire population) PFS (gBRCAm/HRDp/entire population) CFI/TFST/ TSST^h^/ PFS2^i^	TEAEs TEAEs leading to treatment discontinuation TEAEs of anaemia/ leukopenia/ thrombocytopenia/ neutropenia	77(open)	NCT01968213
				Placebo	189	62 (53–68)				
Pujade-Lauraine et al.	2017	SOLO2/ENGOT-Ov21	International, multicenter	Olaparib 300 mg twice daily	196	56 (51–63)	OS (gBRCAm/entire population) PFS (gBRCAm/entire population) TFST/TSST/PFS2	TEAEs TEAEs leading to treatment discontinuation TEAEs of anaemia/ leukopenia/ thrombocytopenia/ neutropenia	65.7	NCT01874353
				Placebo	99	56 (49–63)				
Mirza et al.	2016	ENGOT-OV16/NOVA	International, multicenter	(gBRCAm) Niraparib 300 mg once daily	138	57 (36–83)	OS PFS CFI/TFST/PFS2	TEAEs TEAEs leading to treatment discontinuation	75	NCT01847274
				Placebo	65	58 (38–73)				
				(Non-gBRCAm) Niraparib 300 mg once daily	234	63 (33–84)				
				Placebo	116	61 (34–82)				
Ledermann et al.	2012	Study19	International, multicenter	Olaparib 400 mg twice daily	136	58 (21–89)	OS (BRCAm10/entire population) PFS (BRCAm/entire population) TFST/TSST	NR^j^	78	NCT00753545
				Placebo	129	59 (33–84)				

^a^Progression-free survival

^b^germline BRCA mutation

^c^chemotherapy-free interval

^d^treatment-emergent adverse events

^e^overall survival

^f^time to first subsequent therapy or death

^g^homologous recombination deficiency positive

^h^time to second subsequent therapy or death

^i^second progression-free survival

^j^not reported

The risk of bias of the NMA could be found in Supplementary Material Fig. S2.

### PFS

**Fig. 2:**
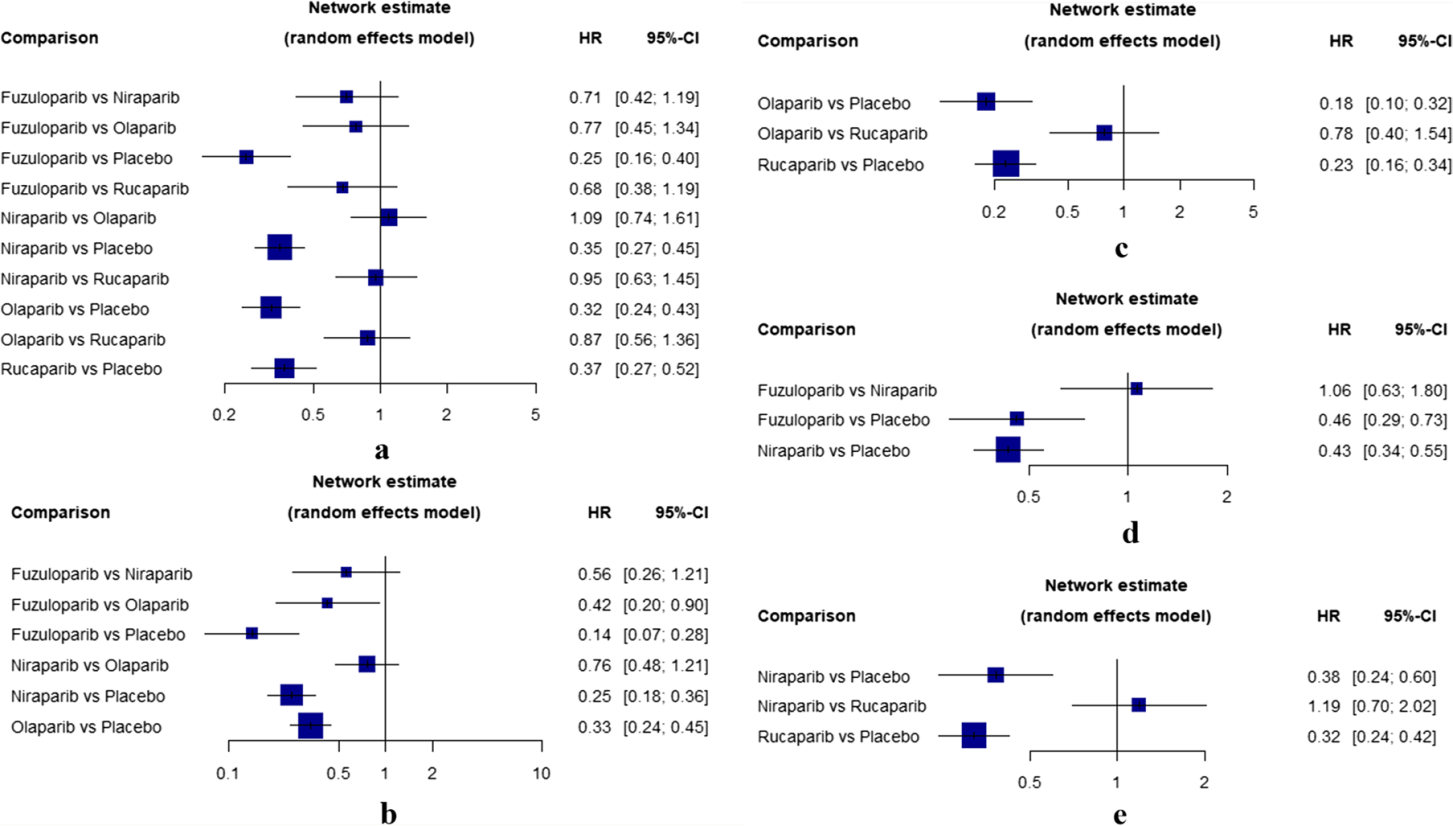
Forest plot of PFS. **a **Forest plot of PFS in entire population. **b** Forest plot of PFS in gBRCA mutated patients. **c** Forest plot of PFS in BRCA mutated patients. **d** Forest plot of PFS in non-gBRCA mutated patients. **e** Forest plot of PFS in HRD positive patients

In this analysis, six RCTs published PFS data, comparing four PARPis. The heterogeneity in PFS evaluation of entire population was moderate with an overall I^2^ statistics of 37.3% and Cochran’s Q test was not significant (Q = 4.79, *P* = 0.1881). Results showed that fuzuloparib (HR, 0.25; 95% CI, 0.16–0.40), olaparib (HR, 0.32; 95% CI, 0.24–0.43), niraparib (HR, 0.35; 95% CI, 0.27–0.45) and rucaparib (HR, 0.37; 95% CI, 0.27–0.52) contributed to a statistically significant PFS benefit (Fig. 2a) compared to placebo. Based on a ranking determined by the NMA that indicates the likehood of providing the greatest PFS benefit, fuzuloparib ranked first (P-score: 90.92%), followed by olaparib (P-score: 64.51%), niraparib (P-score: 50.21%), and rucaparib (P-score: 44.37%). All the treatment rankings in PFS were displayed in Supplementary Material Table S2.

Multiple comparisons of subgroup were shown in Fig. 2b, c, d and e. In the subgroup analysis of gBRCA muted patients, fuzuloparib (HR, 0.14; 95% CI, 0.07–0.28), niraparib (HR, 0.25; 95% CI, 0.18–0.36) and olaparib (HR, 0.33; 95% CI, 0.24–0.45) were significantly effective compared to placebo in PFS, and the corresponding P-score were 97.2%, 64.89% and 37.91%, respectively. For BRCA muted patients, both olaparib (HR, 0.18; 95% CI, 0.10–0.32) and rucaparib (HR, 0.23; 95% CI, 0.16–0.34) were significantly effective in comparison to placebo in PFS, and the corresponding P-scores were 88.01% and 61.99%, respectively. In the subgroup analysis of non-gBRCAm of 3 trials, both niraparib (HR, 0.43; 95% CI, 0.34–0.55) and fuzuloparib (HR, 0.46; 95% CI, 0.29–0.73) had significant benefit in comparison to placebo with P-scores 79.39% and 70.58%, respectively. For HRD positive patients, both rucaparib (HR, 0.32; 95% CI, 0.24–0.42) and niraparib (HR, 0.38; 95% CI, 0.24–0.60) were significantly effective compared to placebo in PFS, and the and the corresponding P-score were 86.88% and 63.12%, respectively.

### OS

**Fig. 3:**
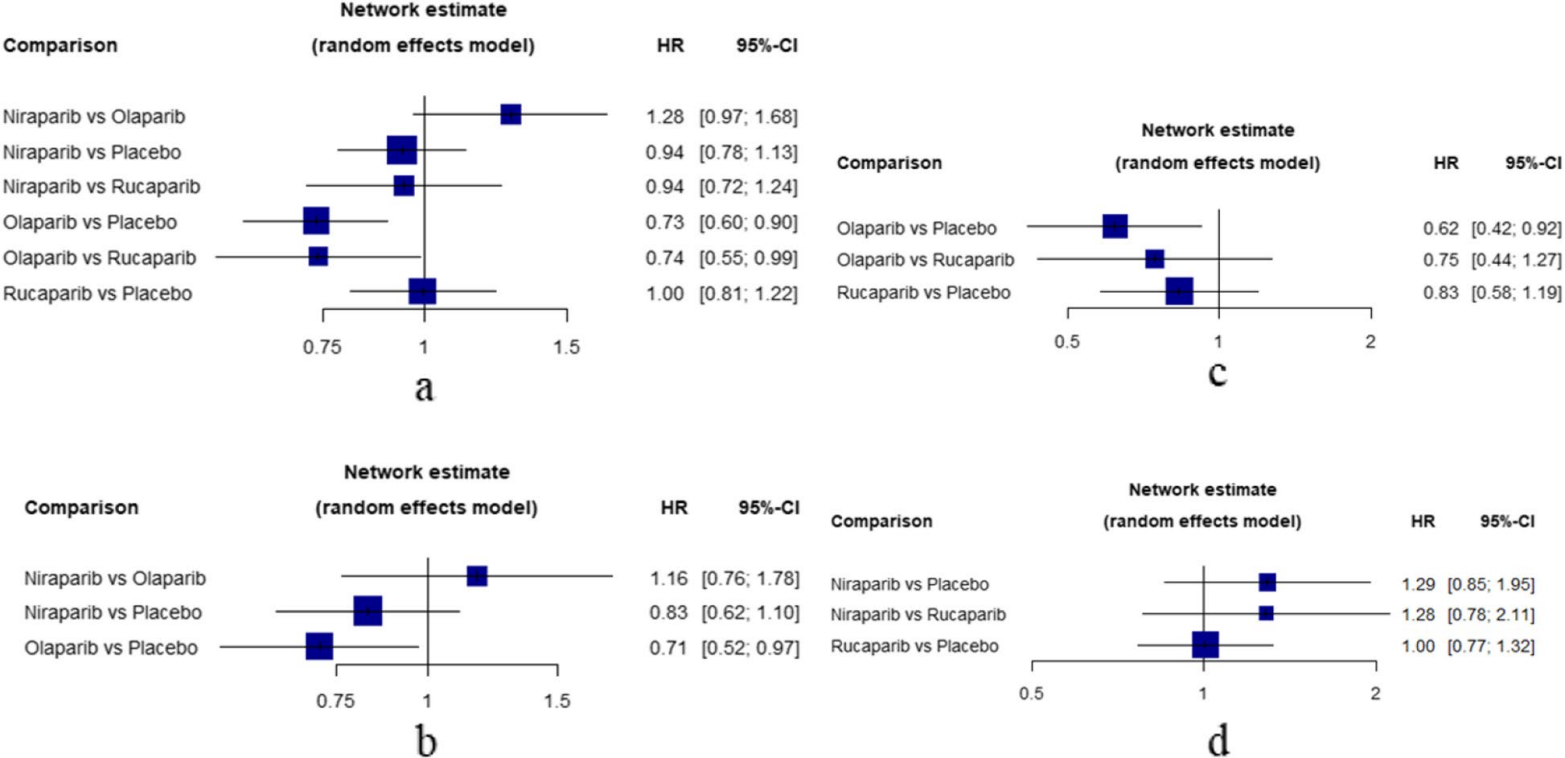
Forest plot of OS. **a** Forest plot of OS in entire population. **b **Forest plot of OS in gBRCA mutated patients. **c** Forest plot of OS in BRCA mutated patients. **d** Forest plot of OS in HRD positive patients

Within this study, there were five RCTs that provided OS data. Out of these trials, two contained groups that were treated with olaparib, two included groups that were treated with niraparib, and one included a group that was treated with rucaparib. The heterogeneity in OS evaluation of entire population was low with an overall I^2^ statistics of 0% and and Cochran’s Q test was not significant (Q = 1.62, *P* = 0.6549). Results of multiple treatment comparisons in the entire population were summarized in Fig. 3a. Olaparib (HR, 0.73; 95% CI, 0.60–0.90) could significantly decrease overall death, but no significant difference was found between niraparib (HR, 0.94; 95% CI, 0.78–1.13) and rucaparib (HR, 1.00; 95% CI, 0.81–1.22), indicating that only olaparib achieved efficacy on OS. The corresponding P-scores for olaparib, niraparib, rucaparib were 97.94%, 48.44%, 29.27% and 24.35%, respectively.

Similarly, olaparib in BRCA mutated patients (HR, 0.62; 95% CI, 0.42–0.92) and gBRCA muted patients (HR, 0.71; 95% CI, 0.52–0.97) were highly effective in comparison to placebo at improving OS, while rucaparib (HR, 0.83; 95% CI, 0.58–1.19) and niraparib (HR, 0.83; 95% CI, 0.62–1.10) didn’t show significance in the subgroup of BRCAm and gBRCAm compared to placebo respectively. For HRD positive patients, both rucaparib (HR, 1.00; 95% CI: 0.77–1.32) and niraparib (HR, 1.29; 95% CI: 0.85–1.95) were not significantly effective in comparison with placebo at improving OS. Results of multiple treatment comparisons of subgroups were summarized in Fig. 3b, c, and d, and treatment rankings in OS were collected in Supplementary Material Table S3.

### Other survival outcomes

In the analysis of CFI, four RCTs reported data and results suggested that fuzuloparib (HR, 0.30; 95% CI, 0.15–0.62), niraparib (HR, 0.36; 95% CI, 0.25–0.53) and rucaparib (HR, 0.43; 95% CI, 0.24–0.76) had significant benefit in comparison to placebo and the P-scores were 81.76%, 67.00% and 51.15%, respectively. The heterogeneity in CFI evaluation was relatively high (I² = 69.4%; Cochran’s Q = 6.53, *P* = 0.0383).

In the analysis of TFST, five RCTs reported data and results suggested that olaparib (HR, 0.38; 95% CI, 0.28–0.52), niraparib (HR, 0.40; 95% CI, 0.31–0.54) and rucaparib (HR, 0.43; 95% CI, 0.29–0.64) had significant benefit compared to placebo and the P-scores were 76.61%, 66.03%, 57.36%, respectively. The heterogeneity in TFST evaluation was relatively high (I² = 55.9%; Cochran’s Q = 6.8, *P* = 0.0786).

In the analysis of TSST, three RCTs reported data and results suggested that olaparib (HR, 0.52; 95% CI, 0.43–0.63) and rucaparib (HR, 0.68; 95% CI, 0.54–0.85) had significant benefits compared to placebo and the P-scores were 98.03% and 51.95%, respectively. The heterogeneity in TSST evaluation was low (I² = 0; Cochran’s Q = 0.04, *P* = 0.8468).

In the analysis of PFS2, three RCTs reported data and results suggested that olaparib (HR, 0.50; 95% CI, 0.34–0.73), rucaparib (HR, 0.70; 95% CI, 0.58–0.85) and niraparib (HR, 0.75; 95% CI, 0.61–0.93) had significant benefit in comparison with placebo and the P-scores were 97.09%, 58.12% and 44.64%, respectively. The heterogeneity in PFS2 evaluation was low (I² = 0; Cochran’s Q = 0.74, *P* = 0.3884).

Results of multiple treatment comparisons in CFI, TFST, TSST and PFS2 were displayed in Supplementary Material Fig. S3 and the rankings were summarized in Supplementary Material Table S4.

### Safety outcomes

In consideration of the tolerability of adverse effects of grades 1–2, all safety outcomes in this study were evaluated based on adverse effects of grades 3–4; the results were illustrated using the ORs and the 95%CIs.

Five trials contributed to the NMA of TEAEs (grade 3–4), comparing the four treatments. The heterogeneity in TEAEs (grade3-4) evaluation was low with an overall I^2^ statistics of 0 and and Cochran’s Q test was not significant (Q = 0.59, *p* = 0.4437). Compared with placebo, olaparib (OR, 2.40; 95% CI, 1.56–3.70), niraparib (OR, 3.06; 95% CI, 2.42–3.87), rucaparib (OR, 3.76; 95% CI, 2.68–5.28), and fuzuloparib (OR, 4.47; 95% CI, 2.36–8.46) led to a numerically higher risk of TEAEs (grade 3–4) and the P-scores were 67.97%, 46.71%, 22.49% and 12.82% respectively. For TEAEs (grade 3–4) leading to treatment discontinuation, niraparib, fuzuloparib, olaparib and rucaparib were included in the NMA and none of them demonstrated significant difference comparing to placebo.

When it comes to severe haematological side effects of grade 3–4 TEAEs, niraparib, olaparib, fuzuloparib, and rucaparib presented significantly higher risks of anaemia when compared with a placebo. Rucaparib and fuzuloparib also presented a significantly higher risk of neutropenia and thrombocytopenia, while olaparib was not significantly different from placebo. Fuzuloparib had a significantly higher risk of leukopenia, whereas olaparib and rucaparib were not significantly different from placebo.

Detailed data of safety outcomes were collected in Supplementary Material Fig. S3 and Table S5.

## Discussion

Based on the mechanism of “synthetically lethality,” PARPis have been utilized in first-line maintenance therapy [35–39] as well as second-line maintenance treatment for ovarian cancer and are gradually being promoted from patients with HRD and BRCAm to all patients. All the PARPis included in the NMA demonstrated significant efficacy in prolonging PFS, PFS2, TFST, TSST and CFI, while only olaparib had significant benefit in the analysis of OS. Olaparib also ranked first compared to niraparib and rucaparib in the evaluation of TFST, TSST and PFS2, while fuzuloparib showed superiority to others in PFS and CFI. In terms of safety outcomes, the incidence of grade 3–4 TEAEs was significantly higher in all PARPis included in analysis compared with placebo, while they did not lead to treatment discontinuation. With respect to haematological grade 3–4 TEAEs, all PARPis had higher incidence in anaemia, while olaparib performed best and was comparable to placebo in thrombocytopenia, leukopenia, and neutropenia.

PFS is one of the primary endpoints assessed for maintenance therapy in gynecologic oncology, and one of its advantages is that it won’t be affected by subsequent treatment regimens [40]. Survival analyses in our NMA showed that the four PARPis demonstrated efficacy in prolonging PFS of PSROC patients regardless of HRD or BRCA status, which is similar to the findings of the NMA in PSROC patients by Xu et al. [15] in 2020 and Wang et al. [16] in 2021. Fuluzolparib revealed more benefit than other PARPis in terms of CFI in entire population and PFS in entire population and gBRCAm, which might attribute to its greater exposure (AUC _0–24 h_) in tumors than in plasma and its potent inhibitory effect on the formation of ADP-ribose polymers. However, PFS could not be totally used as surrogate outcome of OS [41], and OS is still the gold standard for tumor therapy assessment. The updated meta-analyses [42, 43] in recent two years showed the significant benefit of PARPis of all muted types in prolonging PFS of maintenance therapy in ROC but no significant benefit from OS. Our NMA compared the OS of olaparib, niraparib and rucaparib of five trials. Results indicated that only PFS benefit of olaparib translated into OS benefit, while the other two medications did not exhibit a significant difference when compared with placebo, which was consistent in BRCAm and gBRCAm subgroups. Future research should further explore whether olaparib can improve OS in BRCA wild-type patients, which could help expand its indications to a broader patient population. The analytical result of OS is different from Wang et al. in 2021, that both olaparib and rucaparib could significantly decrease the risk of death in gBRCAm patients, whose data were relatively insufficient and immature at the timepoint of analysis and the original data were adjusted by authors with the inverse probability weighting method. The NMA conducted by Zhou et al. [44] also found that only olaparib could significantly prolong OS of PRSOC in BRCA muted and wild types, despite that OS results of some trials like NORA and NOVA have not been reported or updated at the time cut-off of their study. OS analyzed in our NMA were applied with original ITT population data, which were reliable with 72.7% maturity for ARIEL3 [26], 44% for NORA [24], 97.6% for NOVA [32], 61% for SOLO2 [30] and 77% for Study19 [34].

Research found that the differences in the efficacy and safety profiles of PARPis might be attributed to their variations in DNA repair inhibition, apoptosis induction, protein phosphorylation, PARP trapping ability, off-target kinase activity, and binding affinities to different PARP family members [45]. Fuluzolparib, niraparib, and rucaparib demonstrated significant effectiveness in terms of other survival outcomes, specifically in the context of CFI. This suggests that patients with PSROC who received PARPis were able to extend the time between chemotherapy cycles, giving them longer time to recuperate from the adverse reaction of previous chemotherapy. In other words, the prolonged CFI may enhance patients’ quality of life and overall treatment tolerability in real-world settings. PFS2 was significantly prolonged for olaparib, rucaparib and niraparib included in the evaluation, suggesting that PARPis did not deprive patients of another opportunity to benefit from subsequent therapy [46]. TFST and TSST, which are clinically meaningful outcomes evaluating disease recurrence as well as reinitiation of the first and second anti-tumor therapies [46], means that olaparib has the most sustained PFS benefit and the most significant survival advantage for PRSOC patients. TSST could also be approximated as PFS2 and is particularly useful in case PFS could not be assessed regularly [41]. Furthermore, TFST and TSST provide real-world insights into how well PARPis delay disease progression and reduce the need for additional therapeutic interventions, both of which are critical factors in patient management.

Our study found that despite the higher TEAEs of grade 3–4, discontinuation of treatment led by them was not significantly different from placebo group. Cecere [47] et al. and István et al. [42] concluded that adverse events are quite similar in PARPis and most of them are manageable with dose reductions and dose interruptions, among which only minority of cases were required to discontinue treatment. Chronic hematological toxicity may lead to aggravated infections, fatigue, and prolonged bleeding time in cancer patients [48], impacting their quality of life and interfering with treatment. With respect to haematological grade 3–4 TEAEs, the incidence of leukopenia with PARPis was comparable to placebo and anaemia was significantly higher with four PARPis compared to placebo. For thrombocytopenia and neutropenia, no significant difference was found between olaparib and placebo, and rucaparib and fuzulopairb were higher than placebo. Among the four PARPis included in safety analysis, olaparib indicated least toxicity among others, which might be one reason for its superiority in OS and other survival outcomes. Studies of Lafargue et al. [49–51] found that haematological toxicity usually occurs early in treatment and stabilizes after a few months, with anaemia being the most common, followed by neutropenia and thrombocytopenia. The exact mechanism of PARPis causing anaemia is not fully understood, and one explanation is that PARP-2 inhibition leads to inhibition of erythropoiesis [52]. Considering frequency of haematological adverse effects, weekly monitoring of total blood counts is recommended for the first month of treatment, monthly for the first year and thereafter periodically until healed [53]. In spite of potential safety concerns, analyses of health-related quality of life (HRQoL) of the included studies suggested that toxicity of PARPis were well tolerated with limited impact even improvement on HRQoL [23, 29, 54–56].

### Implications for practice and future research

Rucaparib, olaparib, niraparib, and fuzuloparib showed their benefit in PFS and other survival outcomes, providing a variety of effective options for clinicians. The unique advantage of olaparib in prolonging survival suggested that it might be preferred for PSROC patients, and future research should focus on the specific mechanisms for more in-depth theoretical support. Although we found that the efficacy of PARPis in PFS was not affected by BRCA and HRD mutation status, further analyses on different mutation types are needed to guide clinical practice more precisely. The safety profile helps to enhance confidence in promoting PARPis, but long-term monitoring and evaluation of safety are still required. Besides, with the widespread use of PARPi in first-line treatment of ovarian cancer, whether benefit continues in second or later line treatment needs further investigation.

### Strengths and weaknesses

This is the first NMA that directly and comprehensively compare the long-term effectiveness of four PARPis (olaparib, niraparib, rucaparib and fuzuloparib) of monotherapy maintenance treatment in PSROC patients in terms of prolonging survival including OS, PFS, PFS2, TFST, TSST and CFI, as well as TEAEs in grades 3–4 of all kinds, TEAEs (grade 3–4) leading to discontinuation, and haematological TEAEs (grade 3–4). The NMA incorporated six RCTs, all of which were published in prestigious medical publications known for their excellent quality. Nevertheless, the quantity of experiments that have been included is limited and needs additional supplementation. This NMA was carried out at the research level and might not account for confounding factors that exist at the individual patient level. Some of the trials included in the study did not record the HRD status, which could introduce bias. Despite conducting a thorough search of the existing literature based on specific criteria for inclusion and exclusion, there remains a possibility of overlooking certain studies, particularly those that have not been made publicly available or published in English or Chinese. This could potentially introduce publication bias. The limited clinical observation indices and brief follow-up duration in particular research may have a discernible influence on the study outcomes.

## Conclusion

The PARPi represents a significant advancement in managing ovarian cancer. Our study has verified the substantial effectiveness of PARPis in patients with PSROC, in terms of prolonging PFS. This effect is observed regardless of the patient’s BRCA and HRD status. Additionally, PARPis have shown other survival benefits, including PFS2, TFST, TSST, and CFI. However, only olaparib has exhibited a significant improvement in OS for patients, regardless of their BRCA mutation status. Regarding safety, whereas PARPis exhibited a higher occurrence of grade 3–4 TEAEs and partially haematological TEAEs, they did not frequently result in drug discontinuation. This indicates that PARPis have a tolerable safety profile.

## Supplementary Information

Supplementary Material 1.

## Data Availability

No datasets were generated or analysed during the current study.
