# Supplementary material for: A comprehensive comparison of PARP inhibitors as maintenance therapy in platinum-sensitive recurrent ovarian cancer: a systematic review and network meta-analysis
Source: J Ovarian Res. 2025 Jan 30;18:18. doi: 10.1186/s13048-025-01599-1 (PMC11780803; doi:10.1186/s13048-025-01599-1)
Supplement: Supplementary file 1 — Supplementary Material 1. [file 13048_2025_1599_MOESM1_ESM.pdf]

Supplementary Material

A comprehensive comparison of PARP inhibitors as maintenance therapy in platinum-sensitive recurrent ovarian cancer: a systematic review and network meta-analysis

Shiya Ji[1]*, Lu Chen[2†], Yebo Yu[3†], Xupeng Chen[1], Liwen Wei[1], Lili Gou[1], Cheng Shi[1] and Susu Zhuang[1]

1 Department of Health Education, Nanjing Municipal Center for Disease Control and Prevention, Nanjing, China

2 Clinical Medicine College, Yangzhou University, Yangzhou, China.

3 Department of Social Medicine and Health Education, School of Public Health, Peking University, Beijing, China

† These authors have contributed equally to this work

*Corresponding author:

Shiya Ji,

Department of Health Education, Nanjing Municipal Center for Disease Control and Prevention, No.3, Zizhulin Road; 210003, Nanjing, Jiangsu Province of CHINA

E-mail: jishiya@126.com

List of abbreviations

PARP	=	Poly ADP Ribose Polymerase
OS	=	Overall Survival
PFS	=	Progression-free Survival
PFS2	=	Second progression-free survival
TFST	=	Time to First Subsequent Therapy or Death
TFST	=	Time to Second Subsequent Therapy or Death
CFI	=	Chemotherapy-free interval
BRCAm	=	BRCA Mutation
gBRCAm	=	Germline BRCA Mutation
HRDp	=	Homologous recombination deficiency positive
TEAEs	=	Treatment-Emergent Adverse Events
NA	=	Not applicabale
HR	=	Hazard ratio
OR	=	Odds ratio
CI	=	Confidence Interval

Figure and table legends

Table S1. Electronic search strategies.
Table S2. Analysis of treatment ranking in PFS.
Table S3. Analysis of treatment ranking in OS.
Table S4. Analysis of treatment ranking in TFST, TSST, PFS2 and CFI.
Table S5. Analysis of treatment ranking in TEAEs (grade 3-4), TEAEs (grade 3-4) leading to discontinuation, TEAEs (grade 3-4) leading to anaemia, thrombocytopenia, leukopenia and neutropenia.

Figure S1. Network plots of the comparisons for the network meta-analysis.
(A) PFS in entire population;
(B) PFS in BRCA mutated patients;
(C) PFS in gBRCA mutated patients;
(D) PFS in non-gBRCA mutated patients;
(E) PFS in HRD positive patients;
(F) OS in entire population;
(G) OS in BRCA mutated patients;
(H) OS in gBRCA mutated patients;
(I) OS in HRD positive patients;
(J) TFST in entire population;
(K) TSST in entire population;
(L) PFS2 in entire population;
(M) CFI in entire population;
(N) TEAEs (grade 3-4) and TEAEs (grade 3-4) leading to treatment discontinuation;
(O) TEAEs (grade 3-4) of anaemia;
(P) TEAEs (grade 3-4) of leukopenia/ thrombocytopenia/ neutropenia.
Figure S2. Risk of bias summary assessed by ROB 2 tool.
(A) Each risk of bias item for each individual study at the study level of PFS;
(B) Risk of bias items of all included studies indicated as the percentages at the study level of PFS;
(C) Each risk of bias item for each individual study at the study level of PFS;
(D) Risk of bias items of all included studies indicated as the percentages at the study level of PFS.
Figure S3 Forest plot of outcomes.
(A) Forest plot of PFS in entire population;
(B) Forest plot of PFS in BRCA mutated patients;
(C) Forest plot of PFS in gBRCA mutated patients;
(D) Forest plot of PFS in non-gBRCA mutated patients;
(E) Forest plot of PFS in HRD positive patients;
(F) Forest plot of OS in entire population;
(G) Forest plot of OS in BRCA mutated patients;
(H) Forest plot of OS in gBRCA mutated patients;

(I) Forest plot of OS in HRD positive patients;
(J) Forest plot of TFST in entire population;
(K) Forest plot of TSST in entire population;
(L) Forest plot of PFS2 in entire population;
(M) Forest plot of CFI in entire population;
(N) Forest plot of TEAEs (grade 3-4);
(O) Forest plot of TEAEs (grade 3-4) leading to treatment discontinuation;
(P) Forest plot of TEAEs (grade 3-4) of anaemia;
(Q) Forest plot of TEAEs (grade 3-4) of thrombocytopenia;
(R) Forest plot of TEAEs (grade 3-4) of leukopenia;
(S) Forest plot of TEAEs (grade 3-4) of neutropenia.

Figure S4. Pooled pairwise comparisons of PARP inhibitors in the network meta-analysis.

(A) PFS in entire population;
(B) PFS in BRCA mutated patients;
(C) PFS in gBRCA mutated patients;
(D) PFS in non-gBRCA mutated patients;
(E) PFS in HRD positive patients;
(F) OS in entire population;
(G) OS in BRCA mutated patients;
(H) OS in gBRCA mutated patients;
(I) OS in HRD positive patients;
(J) TFST in entire population;
(K) TSST in entire population;
(L) PFS2 in entire population;
(M) CFI in entire population;
(N) TEAEs (grade 3-4);
(O) TEAEs (grade 3-4) leading to treatment discontinuation;
(P) TEAEs (grade 3-4) of anaemia;
(Q) TEAEs (grade 3-4) of thrombocytopenia;
(R) TEAEs (grade 3-4) of leukopenia;
(S) TEAEs (grade 3-4) of neutropenia.

Table S1. Electronic search strategies

**Pubmed**	
**#1**	"Ovarian Neoplasms"[Mesh]
**#2**	"Neoplasm*, Ovarian"[Title/Abstract] OR " Ovarian Neoplasm*"[Title/Abstract] OR "Neoplasm*, Ovary"[Title/Abstract] OR "Ovary Neoplasm*"[Title/Abstract] OR "Cancer*, Ovary"[Title/Abstract] OR "Ovary Cancer*"[Title/Abstract] OR "Cancer*, Ovarian"[Title/Abstract] OR "Ovarian Cancer*"[Title/Abstract] OR "Cancer of Ovary"[Title/Abstract] OR "Cancer of the Ovary"[Title/Abstract]
**#3**	#1 or #2
**#4**	"Poly(ADP-ribose) Polymerase Inhibitors"[Mesh]
**#5**	"Poly(ADP-ribose) Polymerase Inhibitor*"[Title/Abstract] OR "Inhibitors of Poly(ADP-ribose) Polymerase*"[Title/Abstract] OR "Poly(ADP-ribosylation) Inhibitor*"[Title/Abstract] OR "PARP Inhibitor*"[Title/Abstract] OR "Inhibitor*, PARP"[Title/Abstract]
**#6**	"olaparib"[Title/Abstract] OR "AZD 2281"[Title/Abstract] OR "AZD2281"[Title/Abstract] OR "AZD-2281"[Title/Abstract] OR "AZD221"[Title/Abstract] OR "Lynparza"[Title/Abstract]
**#7**	"rucaparib"[Title/Abstract] OR "PF-01367338"[Title/Abstract] OR "Rubraca"[Title/Abstract] OR "AG 014699"[Title/Abstract] OR "AG014699"[Title/Abstract] OR "AG-014699"[Title/Abstract]
**#8**	"niraparib"[Title/Abstract] OR "niraparib hydrochloride"[Title/Abstract] OR "Zejula"[Title/Abstract] OR "MK 4827"[Title/Abstract] OR "MK4827"[Title/Abstract] OR "MK-4827"[Title/Abstract]
**#9**	"fluzoparib"[Title/Abstract] OR "SHR3162"[Title/Abstract]
**#10**	#4 or #5 or #6 or #7 or #8 or #9
**#11**	("randomized controlled trial"[Publication Type] OR "controlled clinical trial"[Publication Type] OR "randomized"[Title/Abstract] OR "placebo"[Title/Abstract] OR "drug therapy"[Mesh Subheading] OR "randomly"[Title/Abstract] OR "trial"[Title/Abstract] OR "groups"[Title/Abstract]) NOT ("animals"[MeSH Terms] NOT "humans"[MeSH Terms])
**#12**	#3 and #10 and #11

Web of Science
**#1** TS=(“Neoplasm*, Ovarian” OR “ Ovarian Neoplasm*” OR “Neoplasm*, Ovary” OR “Ovary Neoplasm*” OR “Cancer*, Ovary” OR “Ovary Cancer*” OR “Cancer*, Ovarian” OR “Ovarian Cancer*” OR “Cancer of Ovary” OR “Cancer of the Ovary”)<br>**#2** TS=("random* controlled trial*" or random* or placebo)<br>**#3** TS=(“Poly(ADP-ribose) Polymerase Inhibitor*” OR “Inhibitors of Poly(ADP-ribose) Polymerase*” OR “Poly(ADP-ribosylation) Inhibitor*” OR “PARP Inhibitor*” OR “Inhibitor*, PARP”)<br>**#4** TS=(“olaparib” OR “AZD 2281” OR “AZD2281” OR “AZD-2281” OR “AZD221” OR “Lynparza” or“rucaparib” OR “PF-01367338” OR “Rubraca” OR “AG 014699” OR “AG014699” OR “AG-014699” or “niraparib” OR “niraparib hydrochloride” OR “Zejula” OR “MK 4827” OR “MK4827” OR “MK-4827” or “fluzoparib” OR “SHR3162”)<br>**#5** #3 or #4<br>**#6** #1 and #2 and #5
**Chochrane libarary**
**#1** MeSH descriptor: [Ovarian Neoplasms] explode all trees<br>**#2** (“Neoplasm*, Ovarian” OR “ Ovarian Neoplasm*” OR “Neoplasm*, Ovary” OR “Ovary Neoplasm*” OR “Cancer*, Ovary” OR “Ovary Cancer*” OR “Cancer*, Ovarian” OR “Ovarian Cancer*” OR “Cancer of Ovary” OR “Cancer of the Ovary”):ti,ab,kw (Word variations have been searched)<br>**#3** #1 OR #2<br>**#4** MeSH descriptor: [Poly(ADP-ribose) Polymerase Inhibitors] explode all trees<br>**#5** (“Poly(ADP-ribose) Polymerase Inhibitor*” OR “Inhibitors of Poly(ADP-ribose) Polymerase*” OR “Poly(ADP-ribosylation) Inhibitor*” OR “PARP Inhibitor*” OR “Inhibitor*, PARP”):ti,ab,kw (Word variations have been searched)<br>**#6** (“olaparib” OR “AZD 2281” OR “AZD2281” OR “AZD-2281” OR “AZD221” OR “Lynparza” OR “rucaparib” OR “PF-01367338” OR “Rubraca” OR “AG 014699” OR “AG014699” OR “AG-014699” OR “niraparib” OR “niraparib hydrochloride” OR “Zejula” OR “MK 4827” OR “MK4827” OR “MK-4827” OR “fluzoparib” OR “SHR3162”):ti,ab,kw (Word variations have been searched)<br>**#7** #4 OR #5 OR #6 1649<br>**#8** #3 AND #7

**Embase**
Query('ovary tumor'/exp OR 'neoplasm of the ovary':ab,ti OR 'neoplasms of the ovary':ab,ti OR 'neoplastic ovarian':ab,ti OR 'neoplastic ovaries':ab,ti OR 'neoplastic ovary':ab,ti OR 'ovarian neoplasia':ab,ti OR 'ovarian neoplasm':ab,ti OR 'ovarian neoplasms':ab,ti OR 'ovarian tumor':ab,ti OR 'ovarian tumorigenesis':ab,ti OR 'ovarian tumour':ab,ti OR 'ovarium tumor':ab,ti OR 'ovarium tumour':ab,ti OR 'ovary neoplasm':ab,ti OR 'ovary tumor treatment':ab,ti OR 'ovary tumorigenesis':ab,ti OR 'ovary tumour':ab,ti OR 'ovary tumour treatment':ab,ti OR 'tumor of the ovary':ab,ti OR 'tumors of the ovary':ab,ti OR 'tumour of the ovary':ab,ti OR 'tumours of the ovary':ab,ti OR 'ovary tumor':ab,ti) AND ('nicotinamide adenine dinucleotide adenosine diphosphate ribosyltransferase inhibitor'/exp OR ((('nad adp ribosyltransferase inhibitor':ab,ti OR 'parp inhibitor':ab,ti OR 'pars inhibitor':ab,ti OR 'poly adp ribose polymerase inhibitor':ab,ti OR 'poly adp ribose synthetase inhibitor':ab,ti OR poly:ab,ti) AND 'adp ribose':ab,ti AND 'polymerase inhibitor*':ab,ti OR poly:ab,ti) AND 'adp ribose':ab,ti AND 'polymerase inhibitors':ab,ti) OR 'nicotinamide adenine dinucleotide adenosine diphosphate ribosyltransferase inhibitor':ab,ti OR 'olaparib'/exp OR 'rucaparib'/exp OR 'niraparib'/exp OR 'fluzoparib'/exp OR 'azd 2281':ab,ti OR 'azd2281':ab,ti OR 'ku 0059436':ab,ti OR 'ku 59436':ab,ti OR 'ku0059436':ab,ti OR 'ku59436':ab,ti OR 'lynparza':ab,ti OR 'mk 7339':ab,ti OR 'mk7339':ab,ti OR 'ng 1002':ab,ti OR 'ng1002':ab,ti OR 'ro 8508245':ab,ti OR 'ro8508245':ab,ti OR 'olaparib':ab,ti OR 'ag 014699':ab,ti OR 'ag 14447':ab,ti OR 'ag 14699':ab,ti OR 'ag014699':ab,ti OR 'ag14447':ab,ti OR 'ag14699':ab,ti OR 'co 338':ab,ti OR 'co338':ab,ti OR 'pf 01367338':ab,ti OR 'pf 1367338':ab,ti OR 'pf 1367338 bw':ab,ti OR 'pf01367338':ab,ti OR 'pf1367338':ab,ti OR 'pf1367338bw':ab,ti OR 'rubraca':ab,ti OR 'rucaparib camphorsulfonate':ab,ti OR 'rucaparib camsilate':ab,ti OR 'rucaparib camsylate':ab,ti OR 'rucaparib phosphate':ab,ti OR 'rucaparib':ab,ti OR 'gsk 3985771':ab,ti OR 'gsk3985771':ab,ti OR 'jnj 64091742':ab,ti OR 'jnj64091742':ab,ti OR 'l 001946812':ab,ti OR 'l001946812':ab,ti OR 'mk 4827':ab,ti OR 'mk4827':ab,ti OR 'niraparib 4 methylbenzenesulfonate':ab,ti OR 'niraparib hydrochloride':ab,ti OR 'niraparib tosilate':ab,ti OR 'niraparib tosylate':ab,ti OR 'zejula':ab,ti OR 'zl 2306':ab,ti OR 'zl2306':ab,ti OR 'niraparib':ab,ti OR 'airuiyi':ab,ti OR 'fluzoparib':ab,ti OR 'hs 10160':ab,ti OR 'hs10160':ab,ti OR 'shr 3162':ab,ti OR 'shr3162':ab,ti OR 'fuzuloparib':ab,ti) AND ('crossover procedure':de OR 'double-blind procedure':de OR 'randomized controlled trial':de OR 'single-blind procedure':de OR random*:de,ab,ti OR factorial*:de,ab,ti OR crossover*:de,ab,ti OR ((cross NEXT/1 over*):de,ab,ti) OR placebo*:de,ab,ti OR ((doubl* NEAR/1 blind*):de,ab,ti) OR ((singl* NEAR/1 blind*):de,ab,ti) OR assign*:de,ab,ti OR allocat*:de,ab,ti OR volunteer*:de,ab,ti)

Table S2. Analysis of treatment ranking in PFS

Treatment	Entire polulation		BRCAm		gBRCAm		Non-gBRCAm		HRDp	
	Rank	P-score	Rank	P-score	Rank	P-score	Rank	P-score	Rank	P-score
Fuzuloparib	1	90.92%	1	88.01%	1	97.20%	NA	NA	NA	NA
Olaparib	2	64.51%	NA	NA	3	37.91%	NA	NA	NA	NA
Niraparib	3	50.21%	NA	NA	2	64.89%	1	79.39%	2	63.12%
Rucaparib	4	44.37%	2	61.99%	NA	NA	2	70.58%	1	86.88%
Placebo	5	0	3	0	4	0	3	0.03%	3	0

Table S3. Analysis of treatment ranking in OS

Treatment	Entire Polulation		BRCAm		gBRCAm		HRDp	
	Rank	P-score	Rank	P-score	Rank	P-score	Rank	P-score
Olaparib	1	97.94%	1	92.49%	1	87.07%	NA	NA
Niraparib	2	48.44%	NA	NA	2	57.30%	3	13.84%
Rucaparib	3	29.27%	2	49.16%	NA	NA	2	66.18%
Placebo	4	24.35%	3	8.35%	3	5.63%	1	69.98%

Table S4. Analysis of treatment ranking in TFST, TSST, PFS2 and CFI

Treatment	TFST		TSST		PFS2		CFI	
	Rank	P-score	Rank	P-score	Rank	P-score	Rank	P-score
Olaparib	1	76.61%	1	98.03%	1	97.09%	NA	NA
Niraparib	2	66.03%	NA	NA	3	44.64%	2	67.00%
Rucaparib	3	57.36%	2	51.95%	2	58.12%	3	51.15%
Fuzuloparib	NA	NA	NA	NA	NA	NA	1	81.76%
Placebo	4	0	3	0.02%	4	0.15%	4	0.09%

Table S5. Analysis of treatment ranking in TEAEs (grade 3-4), TEAEs (grade 3-4) leading to discontinuation, TEAEs (grade 3-4) leading to anaemia, thrombocytopenia, leukopenia and neutropenia.

Treatment	TEAEs (grade 3-4)		TEAEs (grade 3-4) leading to discontinuation		TEAEs (grade 3-4) leading to anaemia		TEAEs (grade 3-4) leading to thrombocytopenia		TEAEs (grade 3-4) leading to leukopenia		TEAEs (grade 3-4) leading to neutropenia	
	Rank	P-score	Rank	P-score	Rank	P-score	Rank	P-score	Rank	P-score	Rank	P-score
Placebo	1	100%	1	80.03%	1	99.76%	1	90.41%	1	92.59%	1	94.41%
Olaparib	2	67.97%	4	35.91%	3	50.01%	2	69.75%	3	39.69%	2	67.98%
Niraparib	3	46.71%	2	55.79%	2	62.51%	NA	NA	NA	NA	NA	NA
Rucaparib	4	22.49%	5	25.39%	5	17.89%	3	22.64%	2	46.76%	3	27.12%
Fuzuloparib	5	12.82%	3	52.88%	4	19.83%	4	17.20%	4	20.96%	4	10.50%

Figure S1. Network plots of the comparisons for the network meta-analysis.

(A) PFS in entire population;
(B) PFS in BRCA mutated patients;
(C) PFS in gBRCA mutated patients;
(D) PFS in non-gBRCA mutated patients;
(E) PFS in HRD positive patients;
(F) OS in entire population;
(G) OS in BRCA mutated patients;
(H) OS in gBRCA mutated patients;
(I) OS in HRD positive patients;
(J) TFST in entire population;
(K) TSST in entire population;
(L) PFS2 in entire population;
(M) CFI in entire population;
(N) TEAEs (grade 3-4) and TEAEs (grade 3-4) leading to treatment discontinuation.
(O) TEAEs (grade 3-4) of anaemia
(P) TEAEs (grade 3-4) of leukopenia/ thrombocytopenia/ neutropenia

(A) PFS in entire population;

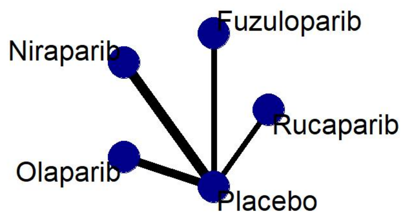

(B) PFS in BRCA mutated patients;

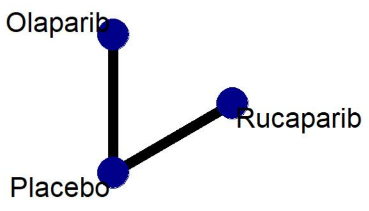

(C) PFS in gBRCA mutated patients;

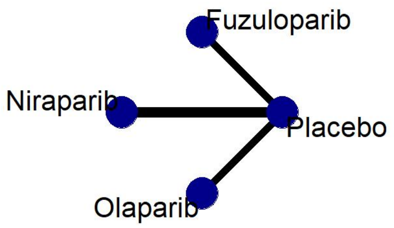

(D) PFS in non-gBRCA mutated patients;

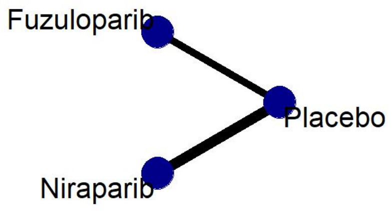

(E) PFS in HRD positive patients;

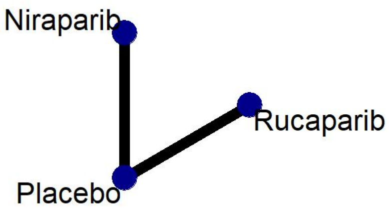

(F) OS in entire population;

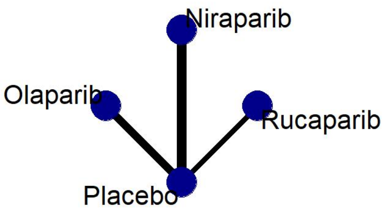

(G) OS in BRCA mutated patients;

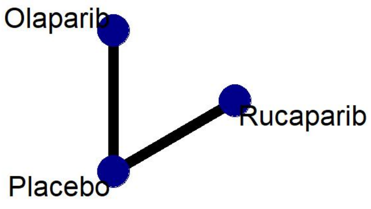

(H) OS in gBRCA mutated patients;

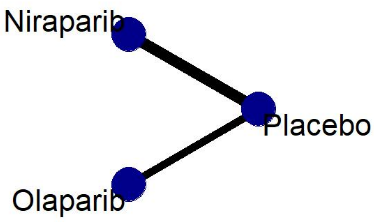

(I)OS in HRD positive patients;

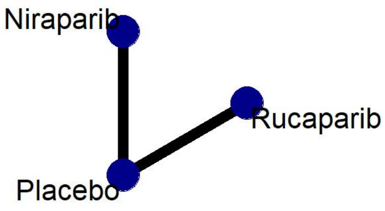

(J)TFST in entire population;

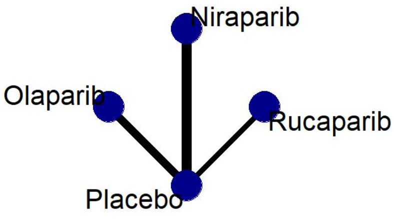

(K) TSST in entire population;

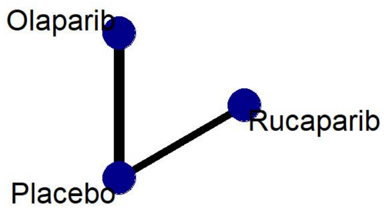

(L) PFS2 in entire population;

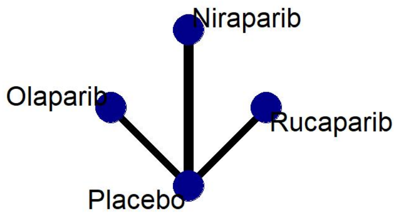

(M) CFI in entire population;

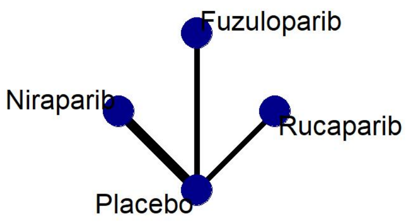

(N) Grade 3-4 (TEAEs) and TEAEs (grade 3-4) leading to treatment discontinuation.

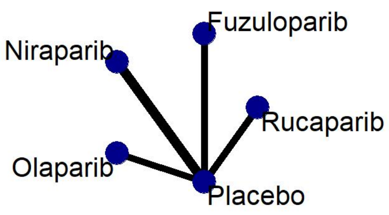

(O) TEAEs (grade 3-4) of anaemia

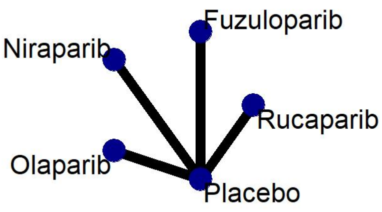

(P)TEAEs (grade 3-4) of leukopenia/ thrombocytopenia/ neutropenia

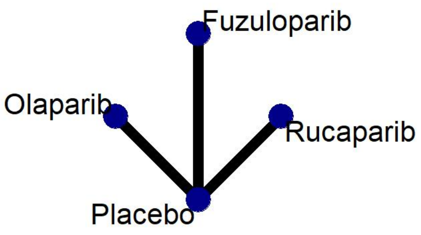

Figure S2. Risk of bias summary assessed by ROB 2 tool

(A) Each risk of bias item for each individual study at the study level of PFS.

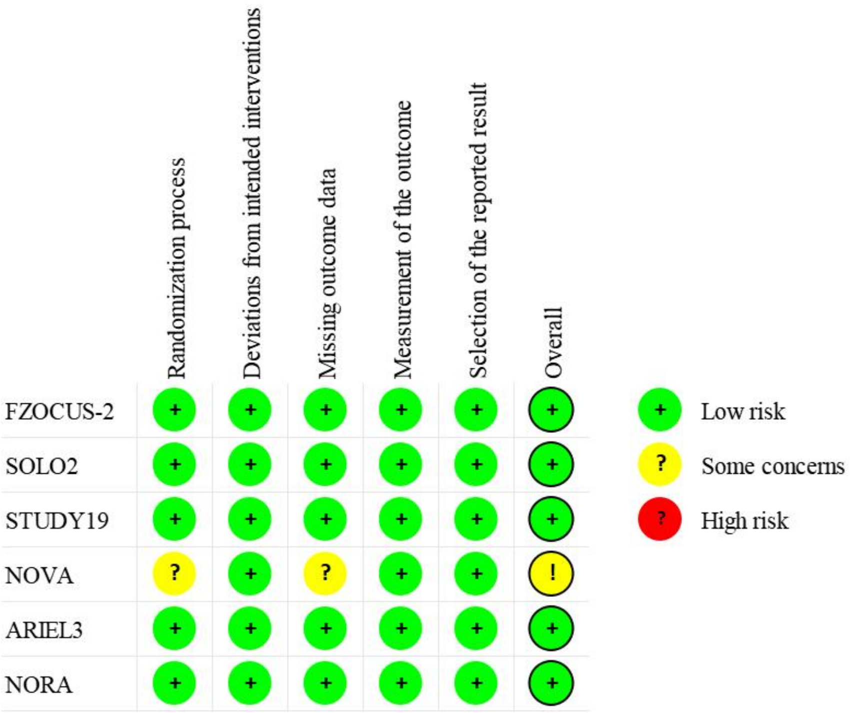

(B) Risk of bias items of all included studies indicated as the percentages at the study level of PFS.

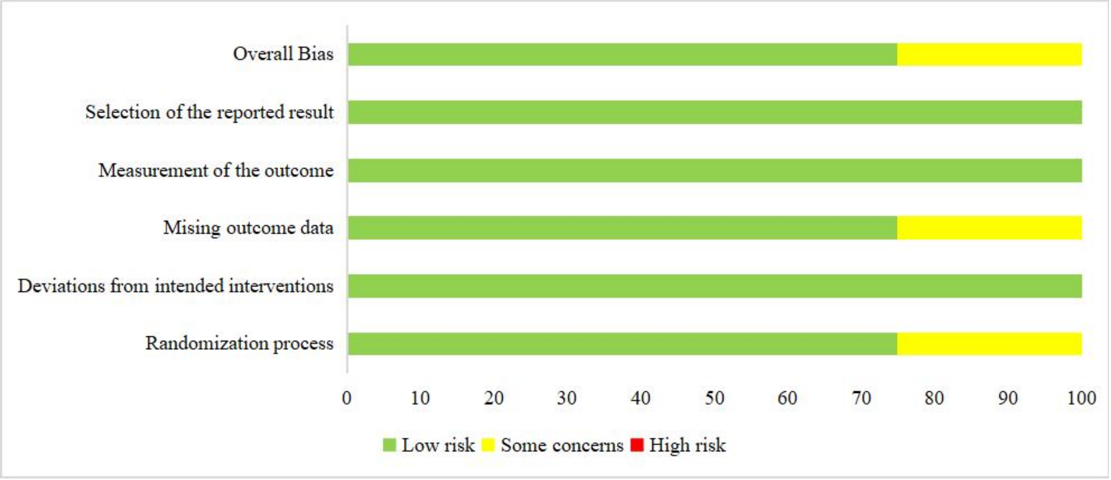

(C) Each risk of bias item for each individual study at the study level of OS.

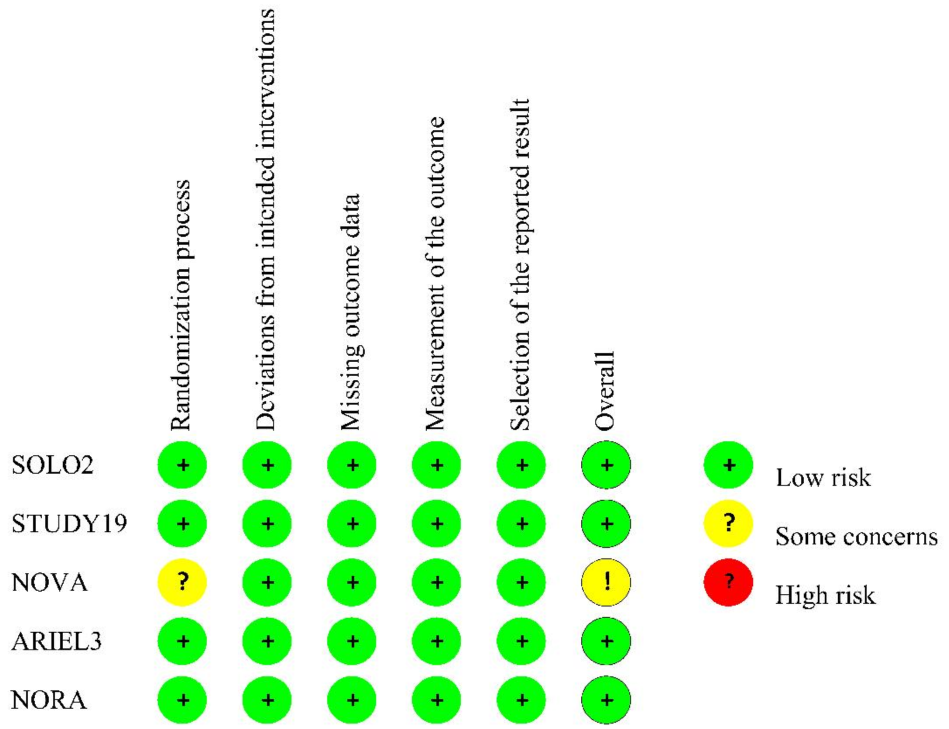

(D) Risk of bias items of all included studies indicated as the percentages at the study level of OS.

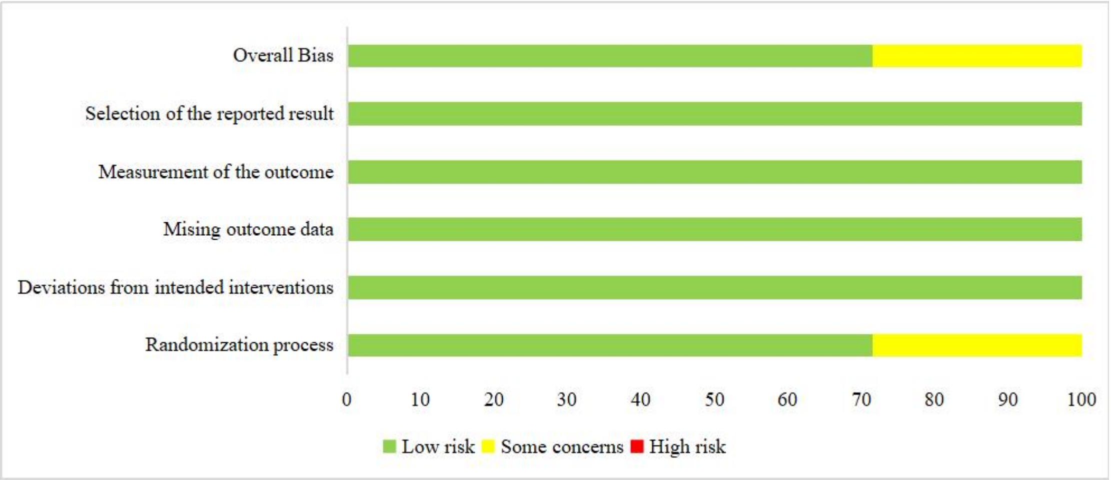

Figure S3 Forest plot of outcomes.

(A) Forest plot of PFS in entire population;

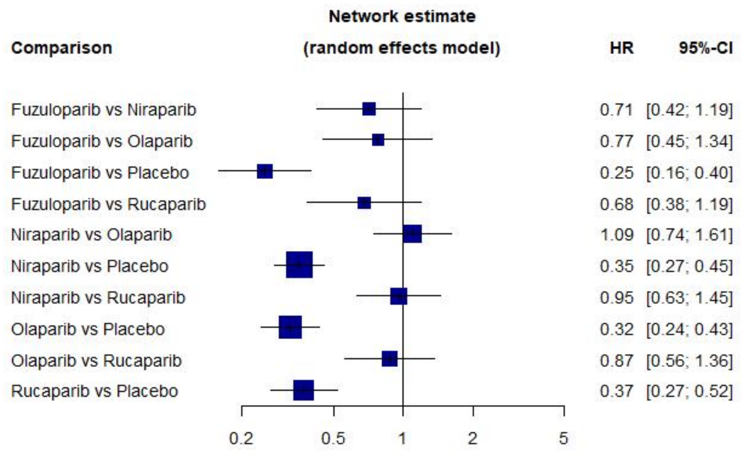

(B) Forest plot of PFS in BRCA mutated patients;

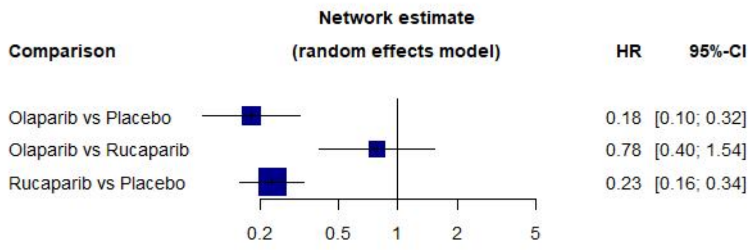

(C) Forest plot of PFS in gBRCA mutated patients;

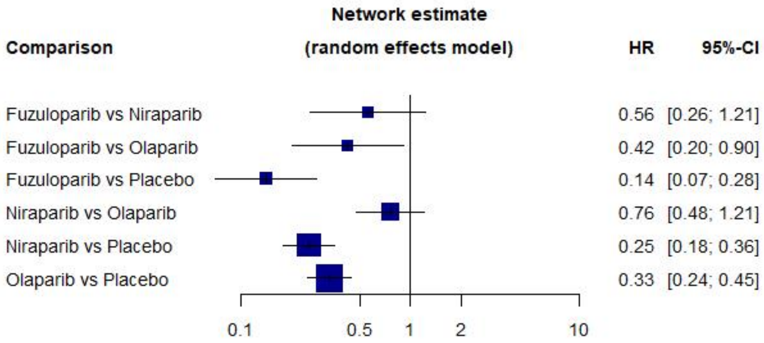

(D) Forest plot of PFS in non-gBRCA mutated patients;

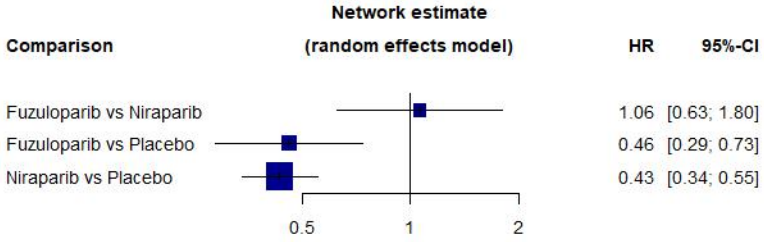

(E) Forest plot of PFS in HRD positive patients;

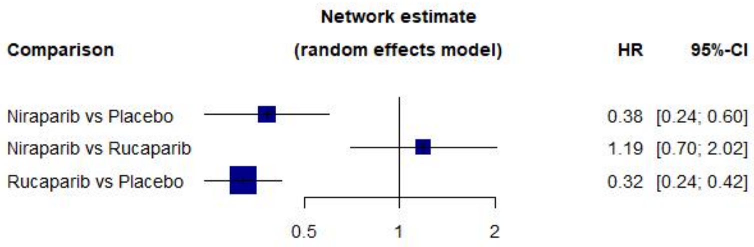

(F) Forest plot of OS in entire population;

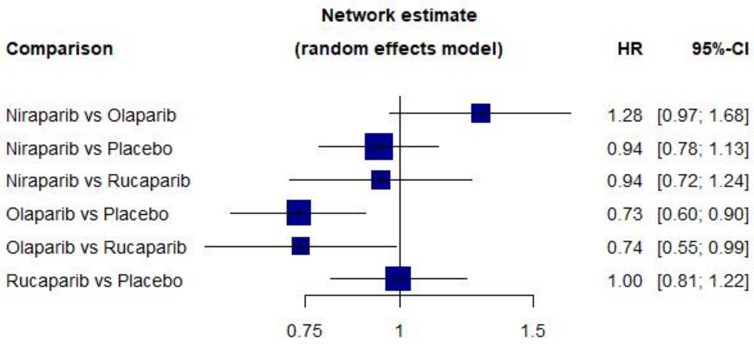

(G) Forest plot of OS in BRCA mutated patients;

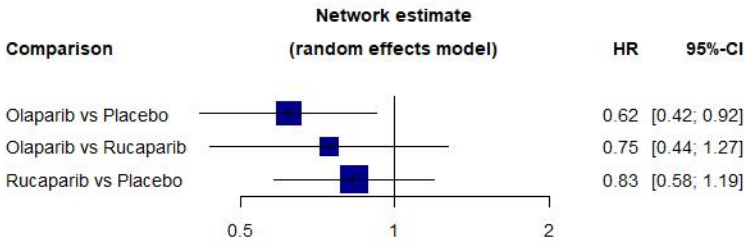

(H) Forest plot of OS in gBRCA mutated patients;

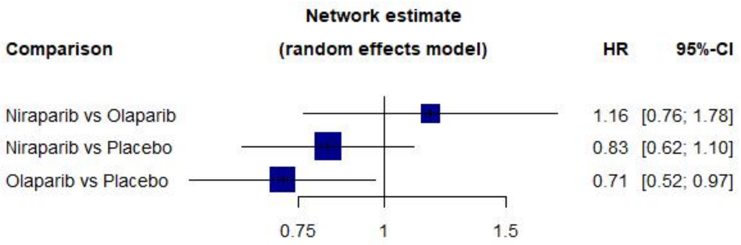

(I) Forest plot of OS in HRD positive patients;

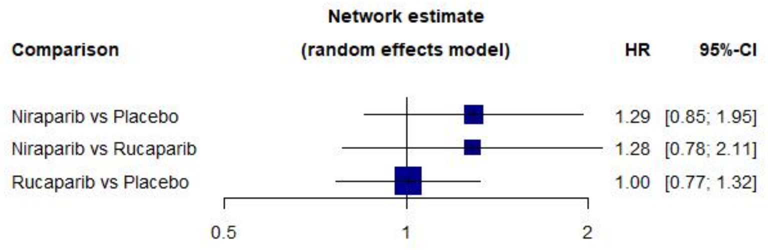

(J) Forest plot of TFST in entire population;

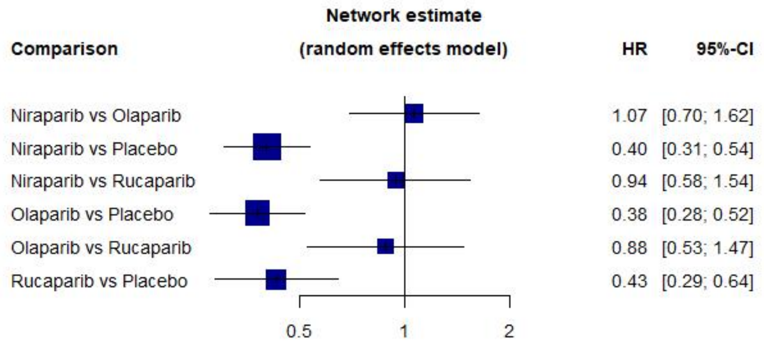

(K) Forest plot of TSST in entire population;

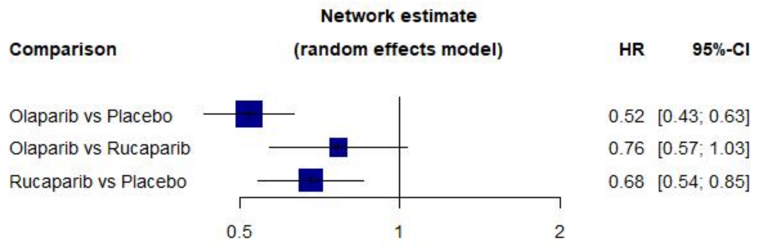

(L) Forest plot of PFS2 in entire population;

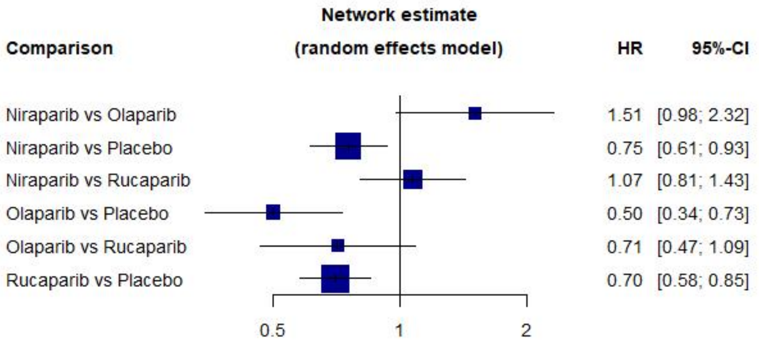

(M) Forest plot of CFI in entire population;

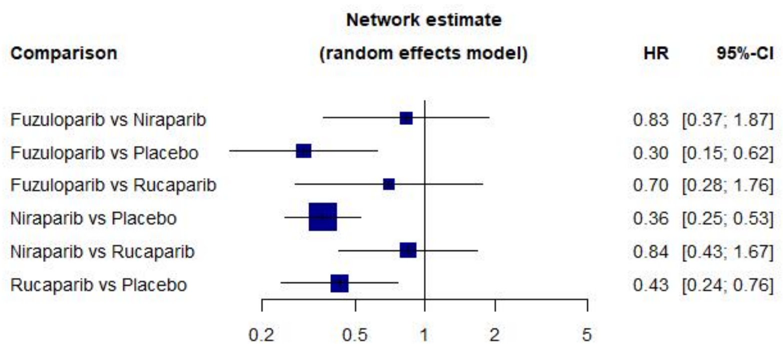

(N) Forest plot of TEAEs (grade 3-4);

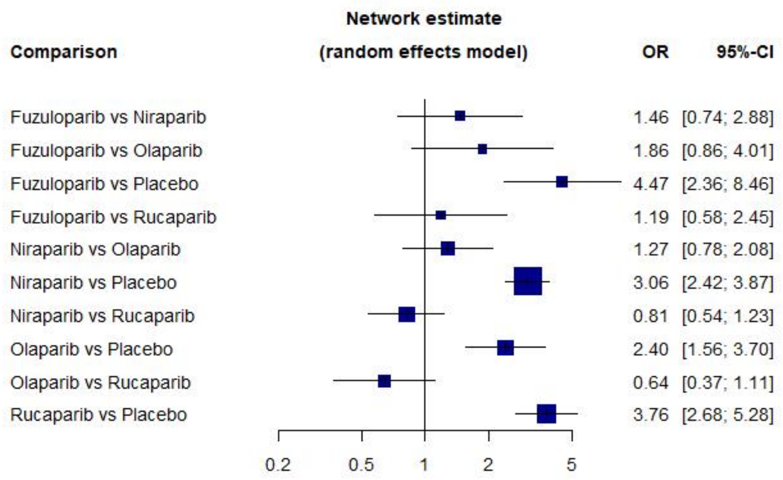

(O) Forest plot of TEAEs (grade 3-4) leading to treatment discontinuation;

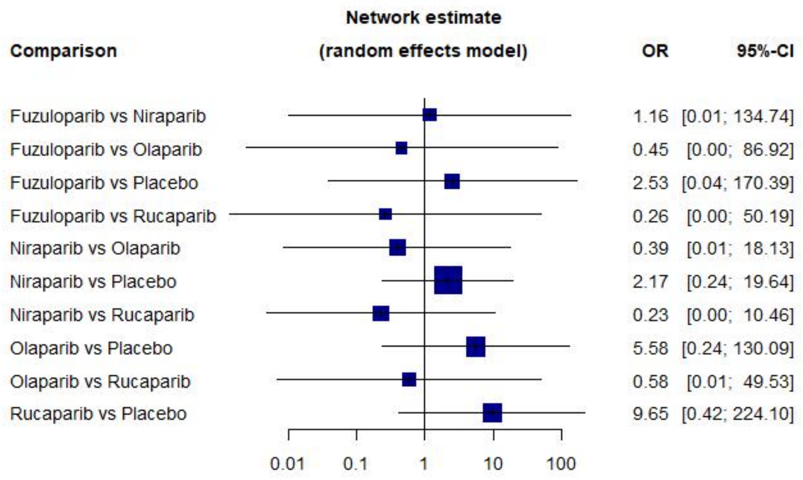

(P) Forest plot of TEAEs (grade 3-4) of anaemia;

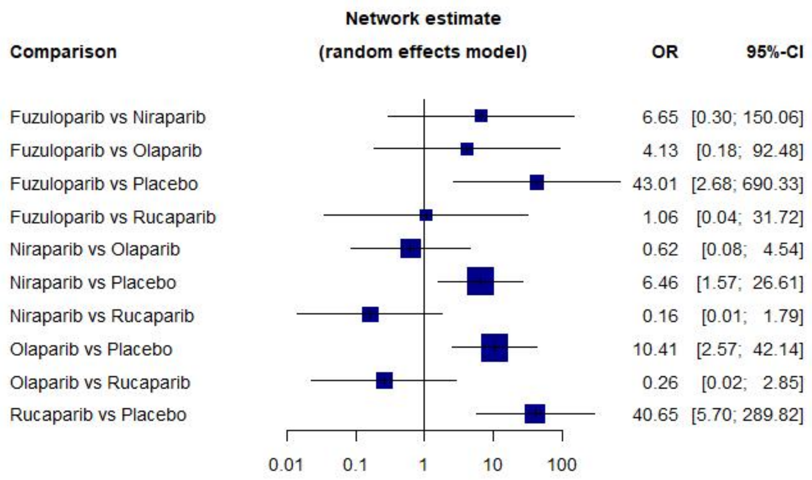

(Q) Forest plot of TEAEs (grade 3-4) of thrombocytopenia;

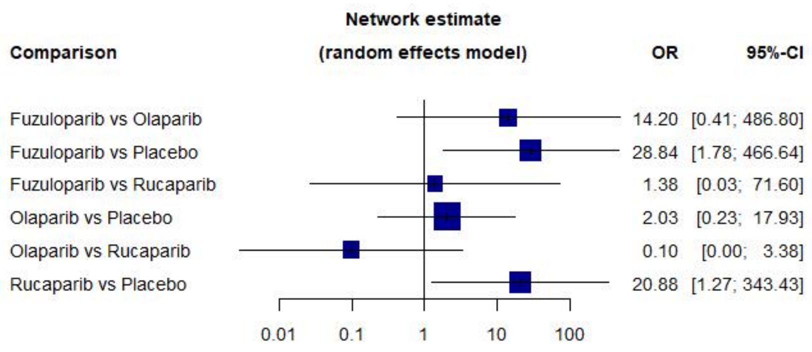

(R) Forest plot of TEAEs (grade 3-4) of leukopenia;

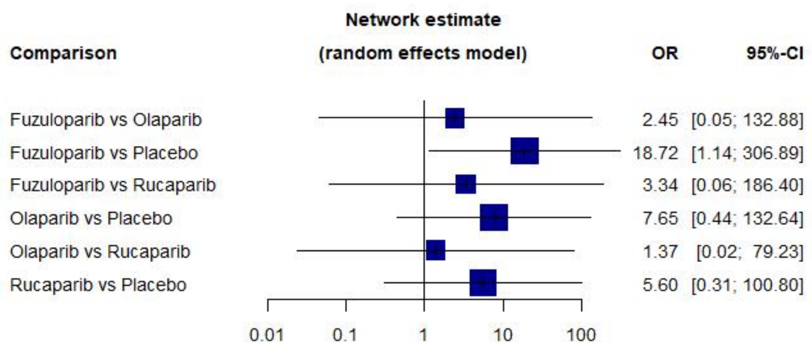

(S) Forest plot of TEAEs (grade 3-4) of neutropenia.

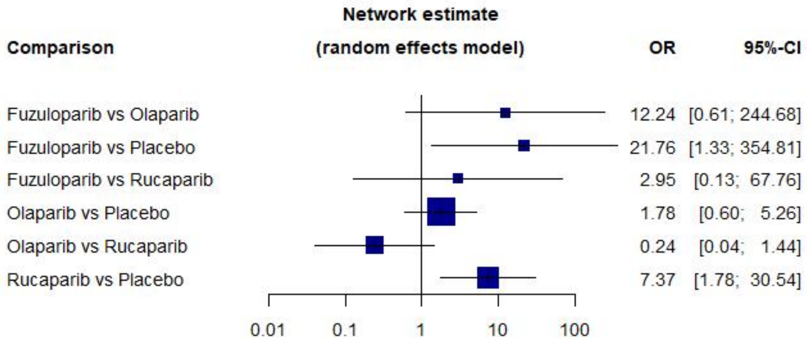

Figure S4 Pooled pairwise comparisons of PARP inhibitors in the network meta-analysis.

Results in each cell represent the pooled HR/OR (HR for survival outcomes and OR for TEAEs) and its 95% CI. The estimates are located at the crossing between the column-defining treatment and row-defining treatment. HR/OR less than one favors the column-defining treatment, and HR/OR more than one favors the row-defining treatment. The significant results are presented in bold.

(A) PFS in entire population;

Fuzuloparib				
0.77 (0.45; 1.34)	Olaparib			
0.71 (0.42; 1.19)	0.91 (0.62; 1.35)	Niraparib		
0.68 (0.38; 1.19)	0.87 (0.56; 1.36)	0.95 (0.63; 1.45)	Rucaparib	
**0.25 (0.16; 0.40)**	**0.32 (0.24; 0.43)**	**0.35 (0.27; 0.45)**	**0.37 (0.27; 0.52)**	Placebo

(B) PFS in BRCA mutated patients;

Olaparib		
0.78 (0.40; 1.54)	Rucaparib	
**0.18 (0.10; 0.32)**	**0.23 (0.16; 0.34)**	Placebo

(C) PFS in gBRCA mutated patients;

Fuzuloparib			
0.56(0.26;1.21)	Niraparib		
**0.42(0.20;0.90)**	0.76(0.48;1.21)	Olaparib	
**0.14(0.07;0.28)**	**0.25(0.18;0.36)**	**0.33(0.24;0.45)**	Placebo

(D) PFS in non-gBRCA mutated patients;

Niraparib		
0.94 (0.56; 1.60)	Fuzuloparib	
**0.43 (0.34; 0.55)**	**0.46 (0.29; 0.73)**	Placebo

(E) PFS in HRD positive patients;

Placebo		
1.00 (0.76; 1.31)	Rucaparib	
0.78 (0.51; 1.17)	0.78 (0.47; 1.28)	Niraparib

(F) OS in entire population;

Olaparib			
0.78 (0.59; 1.03)	Niraparib		
**0.74 (0.55; 0.99)**	0.94 (0.72; 1.24)	Rucaparib	
**0.73 (0.60; 0.90)**	0.94 (0.78; 1.13)	1.00 (0.81; 1.22)	Placebo

(G) OS in BRCA mutated patients;

Olaparib		
0.75 (0.44; 1.27)	Rucaparib	
**0.62 (0.42; 0.92)**	0.83 (0.58; 1.19)	Placebo

(H) OS in gBRCA mutated patients;

Olaparib		
0.86 (0.56; 1.32)	Niraparib	
**0.71 (0.52; 0.97)**	0.83 (0.62; 1.10)	Placebo

(I) OS in HRD positive patients;

Placebo		
1.00 (0.76; 1.31)	Rucaparib	
0.78 (0.51; 1.17)	0.78 (0.47; 1.28)	Niraparib

(J) TFST in entire population;

Olaparib			
0.94 (0.62; 1.43)	Niraparib		
0.88 (0.53; 1.47)	0.94 (0.58; 1.54)	Rucaparib	
**0.38 (0.28; 0.52)**	**0.40 (0.31; 0.54)**	**0.43 (0.29; 0.64)**	Placebo

(K) TSST in entire population;

Olaparib		
0.76 (0.57; 1.03)	Rucaparib	
**0.52 (0.43; 0.63)**	**0.68 (0.54; 0.85)**	Placebo

(L) PFS2 in entire population;

Olaparib			
0.71 (0.47; 1.09)	Rucaparib		
0.66 (0.43; 1.02)	0.93 (0.70; 1.24)	Niraparib	
**0.50 (0.34; 0.73)**	**0.70 (0.58; 0.85)**	**0.75 (0.61; 0.93)**	Placebo

(M) CFI in entire population;

Fuzuloparib			
0.83 (0.37; 1.87)	Niraparib		
0.70 (0.28; 1.76)	0.84 (0.43; 1.67)	Rucaparib	
**0.30 (0.15; 0.62)**	**0.36 (0.25; 0.53)**	**0.43 (0.24; 0.76)**	Placebo

(N) TEAEs (grade 3-4);

Fuzuloparib				
1.46(0.74;2.88)	Niraparib			
1.86(0.86;4.01)	1.27(0.78;2.08)	Olaparib		
1.19(0.58;2.45)	0.81(0.54;1.23)	0.64(0.37;1.11)	Rucaparib	
**4.47(2.36;8.46)**	**3.06(2.24;3.87)**	**2.40(1.56;3.70)**	**3.76(2.68; 5.28)**	Placebo

(O) TEAEs (grade 3-4) leading to treatment discontinuation;

Fuzuloparib				
1.16 (0.01; 134.74)	Niraparib			
0.45 (0.00; 86.92)	0.39 (0.01; 18.13)	Olaparib		
0.26 (0.00; 50.19)	0.23 (0.00; 10.46)	0.58 (0.01; 49.53)	Rucaparib	
2.53 (0.04; 170.39)	2.17 (0.24; 19.64)	5.58 (0.24; 130.09)	9.65 (0.42; 224.10)	Placebo

(P) TEAEs (grade 3-4) of anaemia;

Fuzuloparib				
6.65 (0.30; 150.06)	Niraparib			
4.13 (0.18; 92.48)	0.62 (0.08; 4.54)	Olaparib		
1.06 (0.04; 31.72)	0.16 (0.01; 1.79)	0.26 (0.02; 2.85)	Rucaparib	
**43.01 (2.68; 690.33)**	**6.46 (1.57; 26.61)**	**10.41 (2.57; 42.14)**	**40.65 (5.70; 289.82)**	Placebo

(Q) TEAEs (grade 3-4) of thrombocytopenia;

Fuzuloparib			
14.20 (0.41; 486.80)	Olaparib		
1.38 (0.03; 71.60)	0.10 (0.00; 3.38)	Rucaparib	
**28.84 (1.78; 466.64)**	2.03 (0.23; 17.93)	**20.88 (1.27; 343.43)**	Placebo

(R) TEAEs (grade 3-4) of leukopenia;

Fuzuloparib			
2.45 (0.05; 132.88)	Olaparib		
3.34 (0.06; 186.40)	1.37 (0.02; 79.23)	Rucaparib	
**18.72 (1.14; 306.89)**	7.65 (0.44; 132.64)	5.60 (0.31; 100.80)	Placebo

(S) TEAEs (grade 3-4) of neutropenia.

Fuzuloparib			
12.24 (0.61; 244.68)	Olaparib		
2.95 (0.13; 67.76)	0.24 (0.04; 1.44)	Rucaparib	
**21.76 (1.33; 354.81)**	1.78 (0.60; 5.26)	**7.37 (1.78; 30.54)**	Placebo
